# The Effect of Different Dosages of TESPT on Metal Friction and Metal Wear in the Mixing Process

**DOI:** 10.3390/polym14122314

**Published:** 2022-06-08

**Authors:** Deshang Han, Kongshuo Wang, Chuansheng Wang, Wenwen Han

**Affiliations:** 1 College of Electromechanical Engineering, Qingdao University of Science and Technology, Qingdao 266061, China; handeshang@163.com (D.H.); kongshuo726@163.com (K.W.); wangcs202101@163.com (C.W.); 2 Shandong Provincial Key Laboratory of Polymer Material Advanced Manufactorings Technology, Qingdao University of Science and Technology, Qingdao 266061, China

**Keywords:** TESPT, silica, abrasive wear, corrosive wear

## Abstract

Studies show that the dispersion of silica in the mixing process is an important factor affecting the wear of the mixing chamber. As the most important mixing equipment, the long operational life of the internal mixer will cause wear in the rotor and chamber of the internal mixer. This wear increases the gap between the rotor and chamber of the internal mixer, reduces the mixing performance, weakens the dispersion of packing, and adversely affects the quality of the rubber produced. Therefore, it is important to investigate the metal wear in the mixing process. This article examines the effect of the addition of different amounts of silane coupling agents on metal friction and wear during the mixing process. The silane coupling agent has two functions. The first is to make the surface of the silica hydrophobic, enabling it to combine the inorganic matrix of the silica with the organic matrix of the rubber; the second is to inhibit the aggregation of the silica in the rubber. In the present study, we examine (1) the influence of different formulations on the friction and wear of the metal in the mixing chamber from the perspective of formulation technology, and (2) the correlation between corrosion wear and abrasive wear. It is found that a rubber compound with 6 phr of TESPT has the lowest metal wear and that adding more TESPT does not affect the degree of metal wear. As the amount of TESPT increases, the proportion of abrasive wear decreases, while the proportion of corrosive wear increases, reaching a maximum of 20.7%. In our study we found that abrasive wear is the predominant wear mechanism of a rubber compound on metal. In contrast, the corrosive wear caused by high-temperature water vapor still occupies a large proportion of the total wear. Therefore, improving silica dispersion and reducing abrasive wear are extremely important methods to protect the mixing chamber. However, the corrosion of metals by high-temperature water vapor should also be considered when preparing for the mixing process.

## 1. Introduction

As an important reinforcing filler, silica has been widely used in the rubber industry. Studies show that when used in tires, silica increases wet skid resistance, reduces rolling resistance, and improves traction. However, silica has silanol hydroxyl groups on its surface, resulting in poor compatibility with the main components of tires, including natural rubber, styrene–butadiene rubber, and other nonpolar components. Moreover, the formation of hydrogen bonds between silanol hydroxyl groups can easily affect the composition uniformity of the tire. To resolve this problem, improve the silica dispersion in rubber, and increase the polymer–filler interaction, it is necessary to modify the surface formulation of silica. In this regard, a bifunctional silane coupling agent is usually added to the tire. Reviewing the literature indicates that this issue has attracted scholars worldwide.

Wang Maoying [1] studied the effect of the amount of silane coupling agent on the performance and vulcanization characteristics of silica-filled rubber. As the silane coupling agent increases, the Mooney viscosity of the rubber compound, the Payne effect, and the positive vulcanization time decrease, and the filler dispersion improves. Moreover, the extruding swell ratio of the capillary tube increases, and the extrusion surface deteriorates. Meanwhile, it was found that the tensile stress of the vulcanizate first increases and then decreases.

Yambao Guo [2] studied the effects of different silane coupling agents and hollow glass bead (HGB) filler content on the mechanical and tribological properties of tires under dry friction conditions. Accordingly, it was found that the NBR sample with HGB filler modified by 5 wt.% of silane coupling agent γ-aminopropyl triethoxysilane (KH550) has good interfacial bonding strength and excellent wear resistance. Furthermore, the results revealed that as the amount of KH550 in the tire composition exceeds 5 wt.%, the wear rate of the HGB/NBR composite in the modified HGB sample decreases.

S.S. Sarkawi [3] studied the interaction between silica and natural rubber in the presence and absence of silane coupling agents. In the absence of a silane coupling agent, voids form around the silica particles. On the other hand, the presence of silane results in a strong bond between the filler and the rubber, thereby preventing the formation of cavities. This phenomenon may be attributed to the weak interaction between filler and rubber [4,5].

Kaushik Pal [6] studied the effect of fillers on the rubber’s alloy morphology and wear characteristics. Accordingly, it was found that ISAF N234 significantly improves the tire’s curing, mechanical, and thermal characteristics. The results showed the optimal wear resistance for 80% NR and 20% XNBR with ISAF N234.

It is worth noting that the effects of wet mixing and conventional mixing methods on metal friction and wear have been studied before [7]. More specifically, the corrosion and abrasive wear ratio were quantitatively calculated by spraying high-temperature steam proportionally. Then the wear effect of the two mixing methods on the mixing chamber was simulated.

In the present study, the composition of white carbon black serves as the research object to study the influence of the amount of silane coupling agent on metal friction and wear during the mixing process.

## 2. Experiment

### 2.1. Instrument

In the present study we used a hacker mixer (XSM-500, Qingdao University of Science and Technology, SD, Qingdao, China), a double-roll mill (BL-6157, Dongguan Baolun Precision Testing Instrument Co., SD, Dongguan, China), a CSM-friction-and-wear tester (TRB3, Tribometer Co., Peseux, Switzerland), a steam generator (ZT-2588S, Zhiteng Co., Taiwan, China), a 3D laser measuring microscope (LEXT OLS5000, Olympus, Tokyo, Japan), a rubber processing performance analyzer, (RPA2000, American Alpha Company, Hawthorne, CA, USA), and a dispersion meter (DisperGRADER, American Alpha company, CA, USA).

### 2.2. Chemical Composition

Formulation: The mixing formulation used for the experiments is shown in Table 1.

### 2.3. Mixing Process

Mixing Process: As shown in Table 2.

### 2.4. Testing Method

Test Method:

(1) Payne effect: rubber processing performance analyzer was used to scan deformations on seven rubber compounds. The scanning test was carried out at a scanning frequency of 1 Hz, scanning range of 0.28–40%, and sample temperature of 60 °C. Accordingly, the curve of dynamic modulus Gversus strain was obtained. It is worth noting that the Payne effect originates from the destruction of the network structure between filler and filler. Accordingly, the Payne effect refers to the phenomenon that the dynamic modulus of filled rubber decreases sharply as the strain increases. Generally, the higher the filler aggregation, the worse the dispersibility of the filler, and the more obvious the Payne effect. Therefore, the Payne effect is widely used to reflect the dispersibility of the filler [11,12,13,14,15].
(2) Silylation reaction index: The rubber processing analyzer was utilized to test the silanization reaction index and measure the degree of the silanization reaction. In this regard, the settings are presented in Table 3.

**Figure 1:**
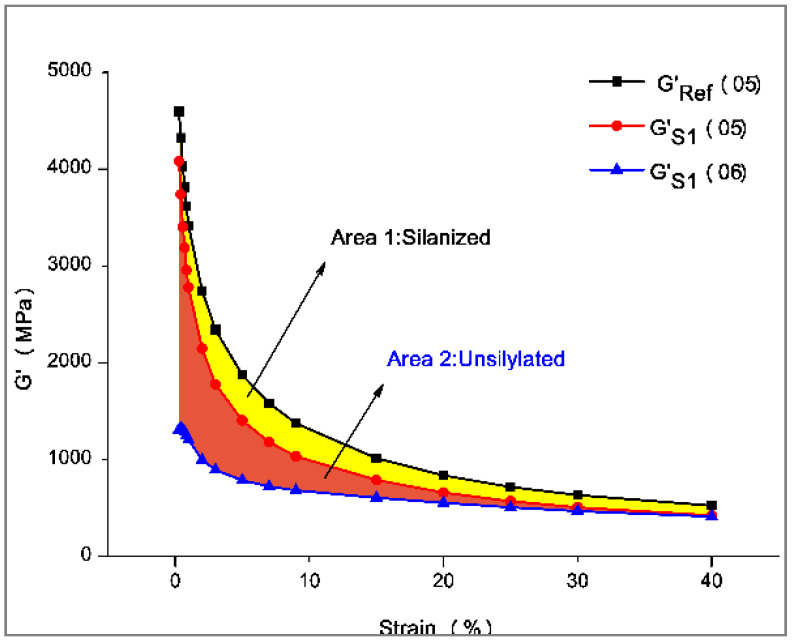
The test principle of silanization reaction degree.

In Stage 1, the sample was preheated. In stages 2 and 3, the filler agglomerations originating from uneven mixing and dispersion were broken. In stage 4, the sample was treated at a constant temperature of 160 °C, which intensifies the polar Brownian motion and reunites the unsilanized filler, thereby increasing the storage modulus [16,17,18,19]. In stage 5, the agglomeration of unsilanized fillers was broken so that $\Delta G^{\prime }(05)$ dropped. Finally, in Stage 6, the entire filler network was broken. If this experiment is to be repeated, a reference value of the sample of the same composition without a coupling agent $\Delta {G^{\prime }}_{REF}(05)$ should be set beforehand. Since the silanization reaction did not occur during the experiment, the filler agglomerated most intensely and the dynamic modulus decreased the most [20]. Figure 1 reveals that the difference between the $\Delta {G^{\prime }}_{REF}(05)$ and the $\Delta G^{\prime }(05)$ distributions is mainly due to the partial silanization of the sample [21,22,23,24,25,26]. If distributions of $\Delta G^{\prime }(05)$ and $\Delta G^{\prime }(06)$ coincide, it means that the sample has reached the maximum degree of silanization. The following expression can be applied in this regard:

(1) $$X=\frac{\mathrm{Area}\;\mathrm{of}\;\mathrm{silylation}\;\mathrm{zoon}}{\mathrm{Area}\;\mathrm{of}\;\mathrm{the}\;\mathrm{largest}\;\mathrm{silylation}\;\mathrm{region}}=\frac{Area1}{Area+Aera2}=\frac{\int {G^{\prime }}_{REF}(05)-\int {G^{\prime }}_{S}(05)}{\int {G^{\prime }}_{REF}(05)-\int {G^{\prime }}_{S}(06)}$$

It should be emphasized that this expression is only applicable for the horizontal comparison.

The silanization reaction index is an important indicator of silica silane modification. The larger the silanization reaction index, the higher the degree of silanization reaction and the better the overall performance of the rubber compound [27,28,29,30,31,32,33,34,35].

**Figure 2:**
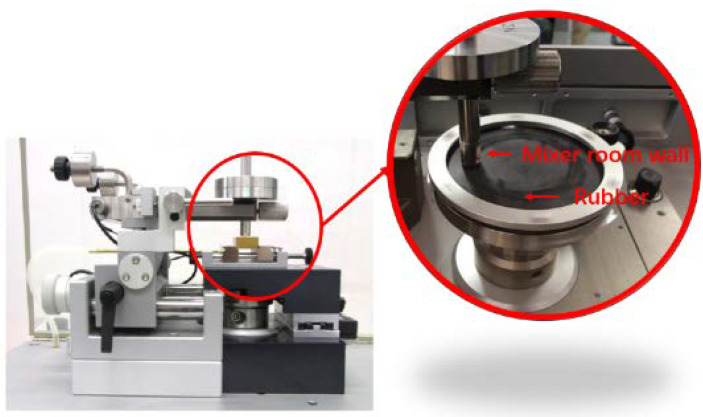
Schematic diagram of the CSM friction experiment.

(3) Friction-and-wear test: A CSM was used in the experiment to carry out the friction- and-wear test. After calibrating the CSM, the pressure, rotating speed, and experiment time were set to 5 N, 80 r/min, and 120 min, respectively. To study the wear of the mixing chamber after long-term use, the selected metal grinding head was not coated. To ensure the authenticity of the experiment, the grinding head and the mixing section were made of the same material. Studies showed that the rubber compound had the most serious wear on the metal in the final stage of the investigation [36,37,38]. Accordingly, the CSM temperature was set to 150 °C. The principal diagram of the CSM wear experiment is shown in Figure 2.
(4) Three-dimensional shape observation: In the present study, a 3D laser measuring microscope was used to observe the surface morphology of the metal and measure the metal wear based on the volume reduction in the metal grinding head [39,40,41,42,43,44,45].
(5) Dispersion test: A dispersion meter was used to test the degree of dispersion and obtain the dispersion value according to the ASTM D7723 standard.

## 3. Silanization Reaction Mechanism

**Figure 3:**
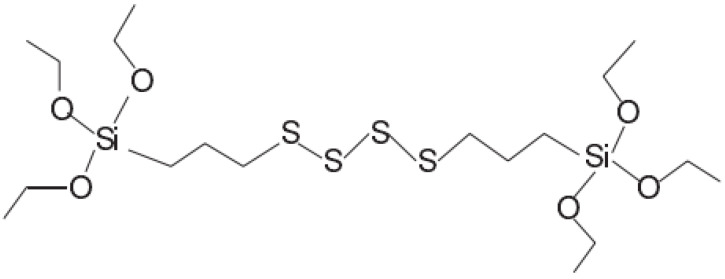
Molecular structure of TESPT.

The full name of TESPT is (bis-(γ-triethoxysilylpropyl)-tetrasulfide). The structure of TESPT is shown in Figure 3 below.

**Figure 4:**
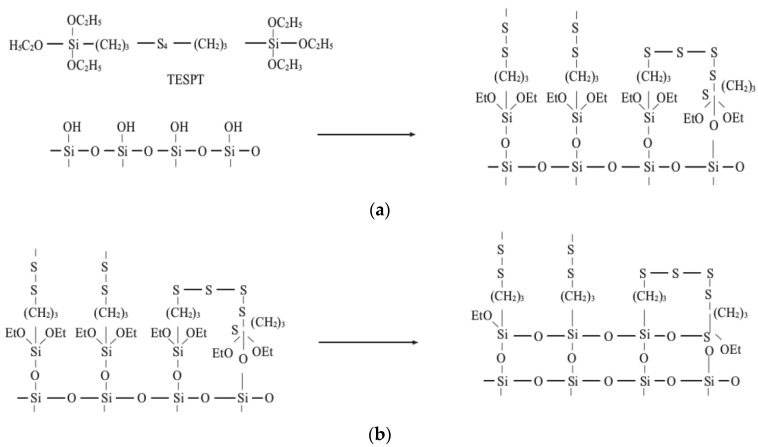
The Silanization Reaction. (**a**) The one-stage reaction of silica with TESPT; (**b**) The two-stage reaction of silica with TESPT.

When TESPT is adsorbed on the silica surface, the surface hydroxyl groups react with the alkoxy groups of the silane in a process called the silanization reaction. The silanization reaction can be divided into two main stages, including the one-stage and two-stage reactions. The one-stage reaction consists of two parts. The first is the direct reaction between the alkoxy group in TESPT and the silanol group on the silica surface (dealcoholization condensation). The second is the dehydration condensation of the alkoxy group of TESPT with the silanol group on the silica surface after decomposition with water. Accordingly, this two-stage reaction can be considered condensation between adjacent TESPTs chemically bonded to the silica surface. The silanization reaction can be expressed as follows (Figure 4):

The silanization reaction process is shown in Figure 4. Studies show that the final process of mixing is more severely worn. The temperature in the final mixing stage is relatively high, and the internal mixer is off. The inner mixing chamber is in a high-temperature environment, and the water vapor cannot overflow the internal mixer. Therefore, the corrosion and wear caused by high-temperature water vapor should be considered in the calculations to study the friction and wear of the inner mixing chamber. However, it is an enormous challenge in the actual process to dismantle the mixing room and measure the quality of the water vapor produced. To resolve this problem, during the friction test on the CSM friction-and-wear tester, high-temperature water was sprayed on the surface of the rubber compound in proportion to the degree of silanization reaction to simulate the mixing situation in the mixing chamber.

## 4. Experiment Results

### 4.1. Filler Dispersion Analysis

#### 4.1.1. Payne Effect

**Figure 5:**
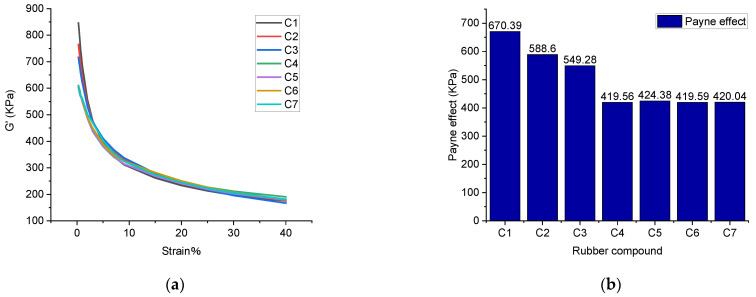
The Payne effect of rubber compounds with different phr of TESPT. (**a**) Stress-strain curve (**b**) Payne effect

The Payne effect is shown in Figure 5.

Figure 5 indicates that the rubber compound without TESPT has the highest Payne effect. As the amount of TESPT increases, the Payne effect of the rubber compound gradually decreases. When the amount of TESPT reaches 6 phr, the Payne effect becomes stable and is no longer affected by TESPT.

It is worth noting that the Payne effect reflects the silica dispersion. Figure 5 reveals that the silica dispersion is worst in the rubber compound without TESPT. Meanwhile, as the amount of TESPT increases, the dispersibility of silica gradually improves. It is found that the best silica dispersibility can be achieved with six TESPT phr, and more TESPT has a negligible effect on the silica dispersion.

#### 4.1.2. Dispersion Comparison

**Figure 6:**
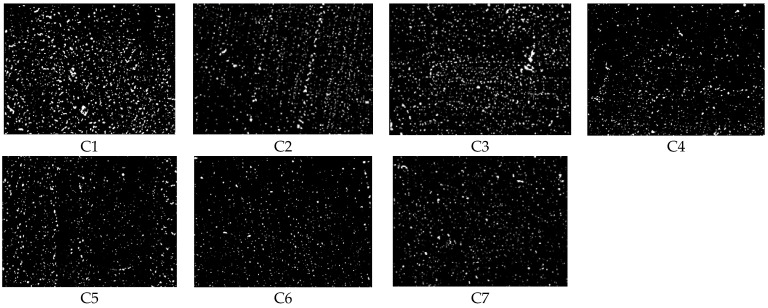
Dispersion image.

Figure 6 is the dispersion image. Table 4 reveals that the silica dispersibility is worst in the rubber compound without TESPT and other large silica aggregates. As the amount of TESPT increases, the dispersion of rubber compounds increases, and the number of silica aggregates decreases. The distribution of rubber compound with 6 TESPT phr is the highest, and the number and volume of silica aggregates in the rubber compound are small. When more TESPTs are added, the rubber compound dispersion changes less, and the number and volume of silica aggregates almost remain constant.

### 4.2. Silanization Reaction Index

**Figure 7:**
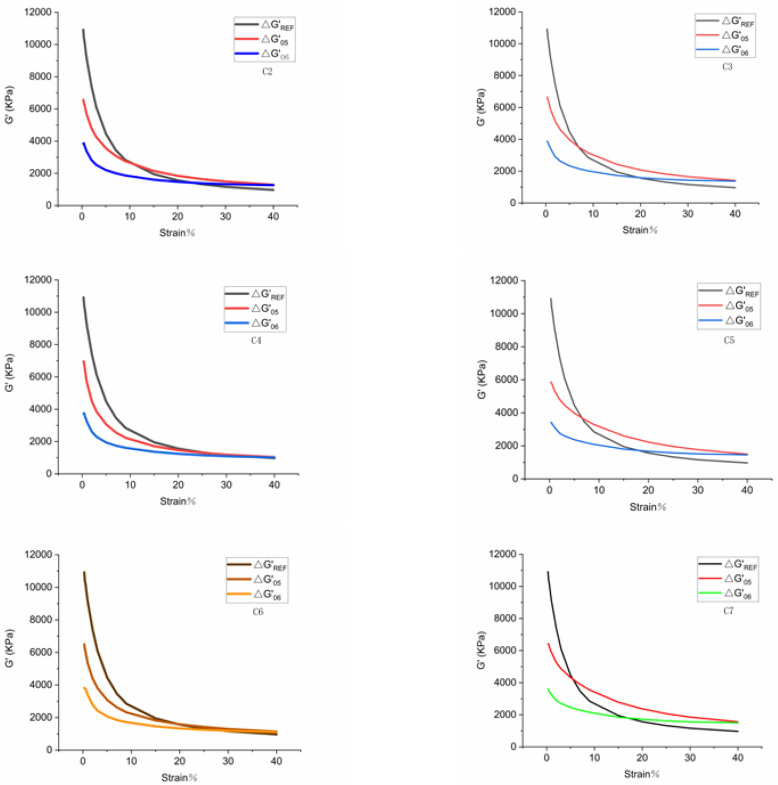
Image of the degree of silanization reaction.

The degree of silanization reaction has an important influence on the performance of the silica rubber compound. The higher the silylation reaction index, the higher the degree of silanization reaction and the more silica and rubber molecules are combined. Moreover, the higher the silanization reaction index, the better the silica dispersion and the better the overall performance of the rubber compound. A rubber processing analyzer was used to obtain the silanization reaction index in the present study. Figure 7 is an image of the extent of the silanization reaction. In this regard, silanization reaction indices of seven rubber compounds are presented in Table 5.

The silanization reaction index reflects the degree of the silanization reaction. The silanization reaction and high-temperature water vapor product have a corrosive effect on metals and can accelerate wear on the metal. However, it is an enormous challenge to dismantle the mixing chamber and measure the quality of the water vapor in the actual process. To resolve this problem, when the friction test was conducted on the CSM friction-and-wear tester, high-temperature water was sprayed on the surface of the rubber compound in proportion to the degree of silanization reaction to simulate the mixing situation in the mixing chamber. Considering the ratio of the silanization reaction index, 150 °C water vapor was sprayed at the ratio of 1:1.88:2.54:3.44:3.36:3.47.

### 4.3. The Effect of Rubber Compounds with Different Amountsof TESPT on Metal Friction and Wear

#### 4.3.1. Friction Coefficient

**Figure 8:**
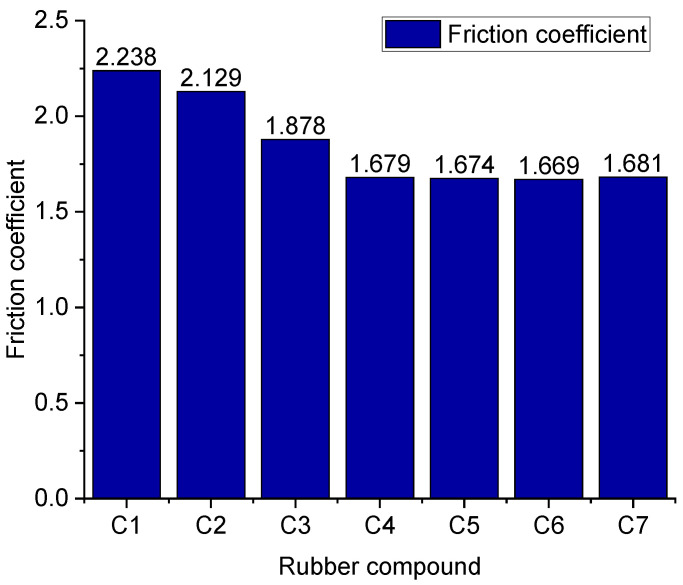
Friction coefficient of the rubber compounds obtained.

Figure 8 reveals that the rubber compound’s friction coefficient correlates with the rubber compound’s dispersion and the proportion of sprayed high-temperature water vapor. The high-temperature water vapor has a lubricating effect on the friction process, reducing the friction coefficient. The better the dispersion of the rubber compound, the lower the friction coefficient. It should be indicated that the rubber compound without TESPT cannot undergo the silanization reaction. The silica molecules easily adsorb each other and form silica aggregates so that the surface of the rubber compound becomes rough and uneven, and the friction coefficient relatively increases. The silanization reaction can occur in the rubber compound with two phr of TESPT. However, since the amount of TESPT is relatively low, silica molecules cannot react completely. This phenomenon leads to more silica that has not undergone silanization reaction in the rubber compound added with 2 phr of TESPT. Since silica has strong mutual adsorption characteristics, many silica aggregates in the rubber compound are added with 2 phr of TESPT. Therefore, compared with the rubber compound without TESPT, the silica aggregate of the rubber compound with 2 phr of TESPT reduces, and the corresponding friction coefficient is also low.

When 4 phr of TESPT are added, more silica molecules participate in the silanization reaction, and the number of silica aggregates greatly reduces. Consequently, the surface of the rubber compound becomes flat so that the friction coefficient further decreases. When 6 phr of TESPT are added, the silanization reaction reaches the maximum. Under this circumstance, the silica reaction is sufficient, and the friction coefficient of the rubber compound reaches the lowest value. When more TESPT is added, the friction coefficient remains constant. From the perspective of the silanization reaction, it is concluded that the maximum silanization reaction under this experimental process can be achieved when 6 phr of TESPT are added. Meanwhile, adding more TESPT does not increase the chemical bond of the silane coupling agent with the silica surface.

#### 4.3.2. Metal Surface Observation

**Figure 9:**
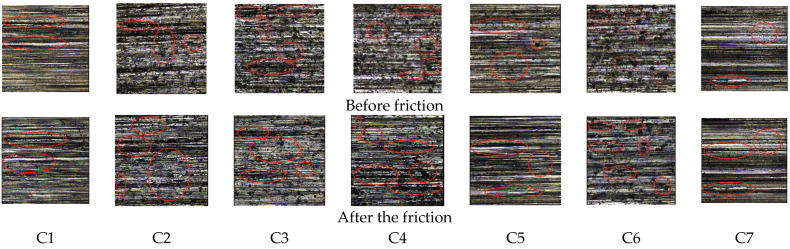
Metal surface morphology before and after friction.

**Figure 10:**
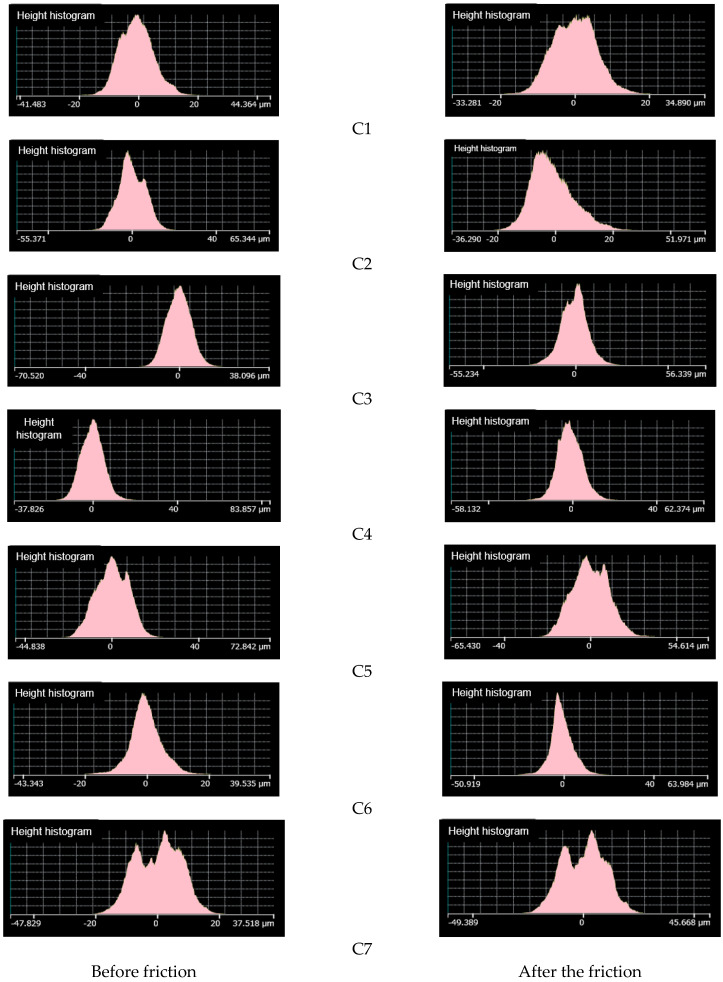
Height histogram.

**Figure 11:**
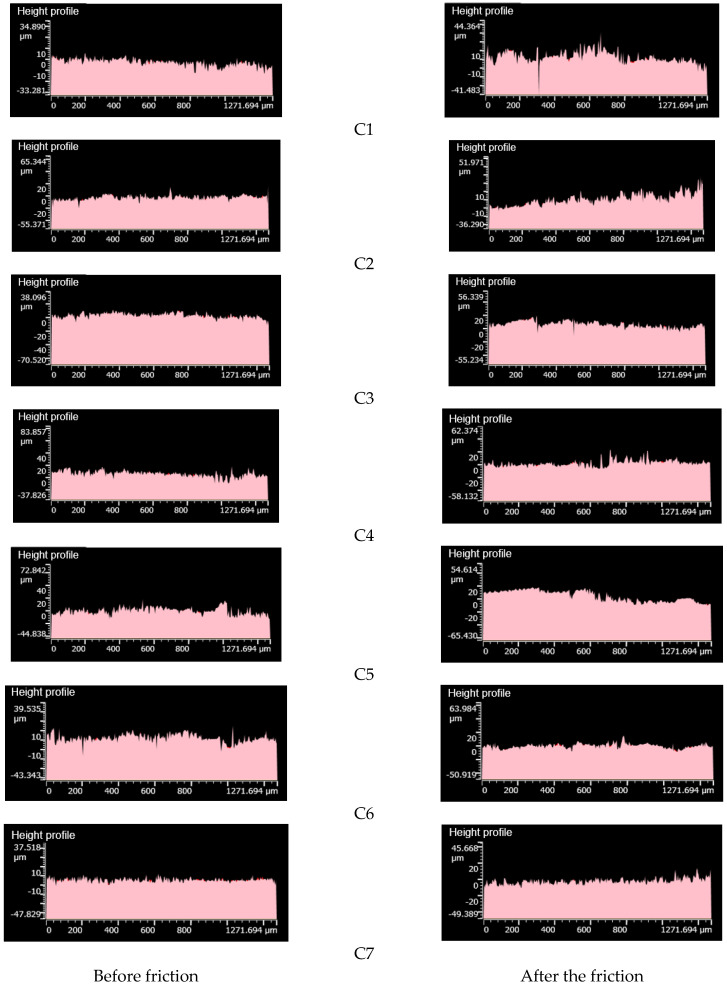
Height profile. C1–C7 are different metal surfaces.

**Figure 12:**
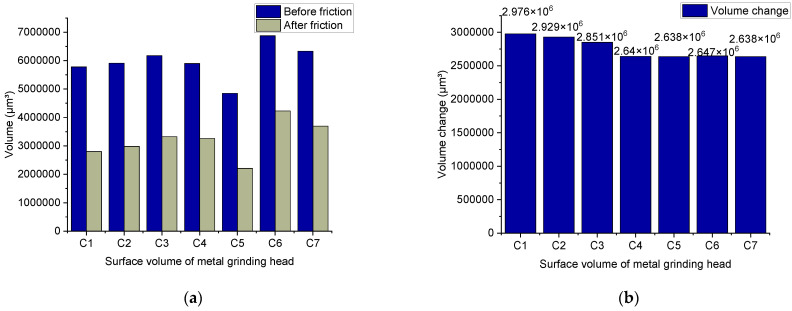
Wear volume of the metal.

Figure 9 is an image of metal surface topography; Figure 10 is a histogram of metal height histogram; Figure 11 is a contour map of metal height profile; and Figure 12 shows metal volume. This can be seen in Figure 9C1, Figure 10C1 and Figure 11C1. After friction, many scratches and pits appeared on the metal surface, and the surface was severely worn. The height histogram changes greatly before and after friction, smoothing the height peaks. The height profile of the metal surface before friction is relatively flat, and it fluctuates greatly after friction. It can be seen from Figure 9C2, Figure 10C2 and Figure 11C2 that there are many scratches and pits on the metal surface after friction. The height histogram changes greatly before and after the comparison friction, and the height peak is smoothed out. The height profile of the metal surface before friction is relatively flat, and the height profile of the metal surface shows an increasing trend after friction. It can be seen from Figure 9C3, Figure 10C3 and Figure 11C3 that there are relatively few scratches on the metal surface after rubbing. The histogram of the height before and after friction is small, and a small height peak appears. The height profile of the metal surface before friction is relatively flat, and the change in the height profile before and after friction is small. It can be seen from Figure 9C4, Figure 10C4 and Figure 11C4 that there are relatively few scratches on the metal surface after friction, and the pits tend to expand. The change in height before and after friction as shown in the histogram is small, but there is a small peak.

The height profile of the metal surface before friction is relatively flat, and the height profile fluctuates greatly after friction. It can be seen from Figure 9C5, Figure 10C5 and Figure 11C5 that there are relatively few scratches on the metal surface after friction, and the pits are smoothed. The height histogram changes obviously before and after friction, and a small peak appears after friction. The height profile of the metal surface fluctuates greatly after friction. It can be seen from Figure 9C6, Figure 10C6 and Figure 11C6 that there are few scratches on the metal surface after friction, and the pits tend to expand. The height profile fluctuates greatly before friction and tends to be flat after friction. It can be seen from Figure 9C7, Figure 10C7 and Figure 11C7 that there are almost no scratches on the metal surface after friction, and the pits tend to expand. The height peak decreases after friction, and the profile changes less before and after friction.

Figure 12 shows the metal wear volume. After the friction-and-wear experiment of the metal grinding head, the average value is obtained after several measurement points. Figure 12 illustrates that the rubber compound without TESPT imposes the highest wear on the metal. However, the wear amount gradually decreases as the silane coupling agent is slowly added. The lowest wear rate is obtained when 6 phr of TESPT is added.

Meanwhile, when more TESPT is added, the value of the metal wear remains constant. It is worth noting that the wear of the silica rubber compound to the metal is not only abrasive but also corrosive. The progress of the silanization reaction is accompanied by water production, and high-temperature water vapor corrodes the metal and accelerates the wear of the metal. The data measured in this group of experiments show the wear volume of the metal under the condition of spraying high-temperature water vapor. In other words, the data consists of the volume of abrasive wear and corrosion wear.

For the rubber compound without TESPT, the silica molecule cannot combine with the rubber molecule because the silanization reaction cannot occur. It is worth noting that the silica molecules adsorb each other to form silica aggregates, causing serious abrasive wear to the metal. With the addition of TESPT, the silanization reaction initiates. When adding 2 phr of TESPT during the mixing process, the silica cannot react completely because the value of TESPT is low. Therefore, there are still many silica aggregates in the rubber compound. As the silanization reaction progresses, high-temperature water vapor is generated, and corrosion wear occurs. In terms of the amount of metal wear, the difference between the rubber compound with 2 phr of TESPT and the rubber compound without TESPT is small. When 4 phr of TESPT are added during the mixing process, more high-temperature water vapor is generated as the silanization reaction proceeds. The high-temperature water vapor corrodes the metal and accelerates the wear of the metal. However, as the silanization reaction progresses, the free silica molecules and the silica aggregates decrease. Therefore, abrasive wear is weakened at this time. By adding 6 phr of TESPT during the mixing process, the silanization reaction proceeds to the maximum.

Meanwhile, the free silica molecules are few, and the number of silica aggregates is low. However, the silanization reaction produces a lot of high-temperature water vapor, which corrodes and accelerates wear on the metal. Therefore, although the abrasive wear is weak at this time, considering the corrosion wear, the wear amount is relatively large. When a larger amount of TESPT is added, the degree of the silanization reaction does not change. Therefore, the amount of high-temperature water vapor generated by the silanization reaction is the same as adding 6 phr of TESPT. During the mixing process, 8, 10, and 12 phr of TESPT are added, and the degree of the silanization reaction is the same as that of 6 phr of TESPT. This also means that the number of silica aggregates in the rubber compound added with 8, 10, and 12 phr of TESPT is less. Therefore, abrasive wear is weakened. Compared with the rubber compound with 6 phr of TESPT, the difference in wear is small.

#### 4.3.3. The Proportion of Corrosion Wear and Abrasive Wear

**Figure 13:**
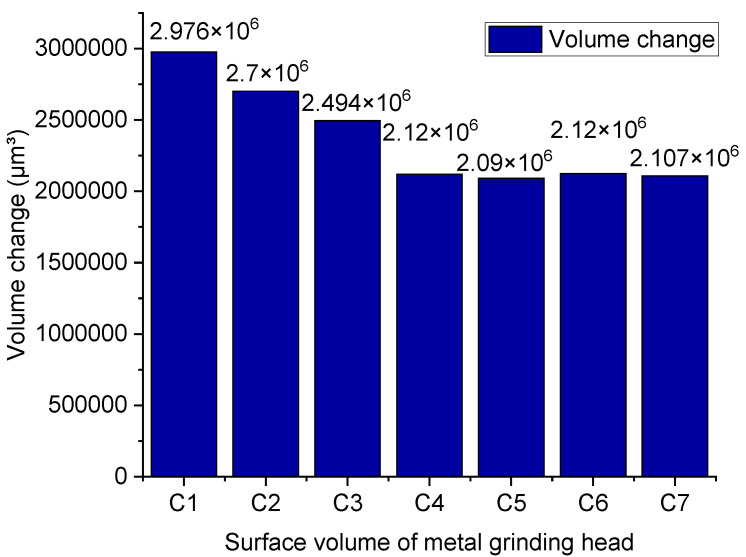
Wear volume of the metal without high-temperature steam spraying.

In this section, another rubber compound without high-temperature steam spraying is considered a control sample, and the CSM friction-and-wear experiment is carried out. Figure 13 shows the volume loss of the metal grinding head after the friction-and-wear test of the rubber compound.

During the experiment, high-temperature water vapor is not sprayed, and metal corrosion will not occur. Meanwhile, the form of the wear is abrasive wear. It should be emphasized that abrasive and corrosive wear occurs when high-temperature water vapor is sprayed. Compare the wear that occurs when high-temperature water vapor is not sprayed at this time. Then, 6 repeated experiments are performed, and the average of the data is considered to calculate the ratio of the abrasive and corrosive wear.

**Figure 14:**
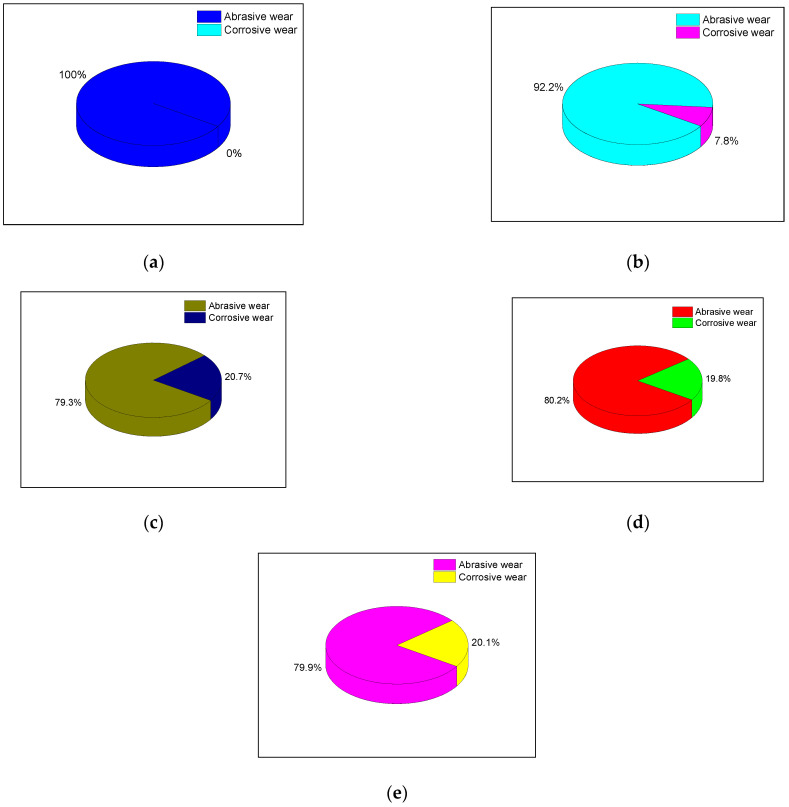
The proportion of abrasive wear and corrosion wear. (**a**–**e**) are wear ratios.

Figure 14 illustrates that as the number of TESPT increases, the proportion of corrosive wear gradually increases. When the number of TESPT phr is 6, the balance of corrosion and wear reaches the maximum value. When more silane coupling agent is added, the proportion of corrosive wear no longer increases. This corresponds to the degree of silanization reaction of the rubber compound. The higher the degree of silanization reaction, the more high-temperature water vapor is produced, and the more serious the corrosion wear of the metals.

#### 4.3.4. Change in Roughness before and after Friction

**Figure 15:**
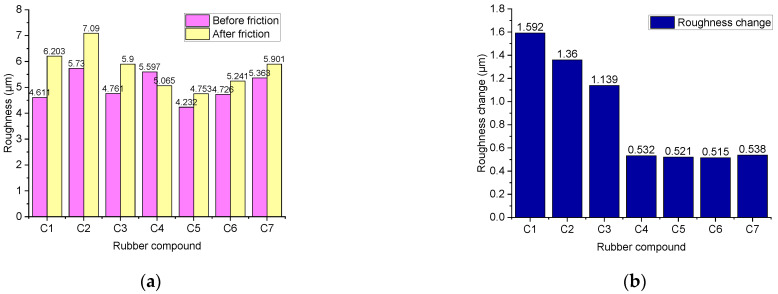
The roughness of the metal surfaces. (**a**) is the metal surface roughness before and after friction, and (**b**) is the roughness change.

Figure 15 shows the change in the roughness of the metal grinding head surface before and after friction. As the number of TESPT increases, the difference in the roughness of the metal surface decreases. With the addition of TESPT and the progress of the silanization reaction, the number of silica aggregates significantly reduces, and abrasive wear reduces too. Therefore, the roughness of the metal surface is reduced. As TESPT increases, the generated high-temperature water vapor increases. Moreover, the high-temperature water vapor plays a lubrication role, which also reduces the variation in the roughness of the metal surface.

## 5. Conclusions

In the present study, we investigated the influence of different formulations on the friction and wear of metal in a mixing chamber. Considering the dispersion and the Payne effect, it is found that as TESPT increases, the degree of the silanization reaction increases, the distribution of silica improves, the number of silica aggregates decreases significantly, and the abrasive wear of the metal is reduced. When 6 phr of TESPT are added, the silanization reaction reaches its maximum, and the silanization reaction index is 0.55879. When the dosage of TESPT increases, the degree of the silanization reaction does not increase; this means that 26.47 silica molecules do not participate in the silanization reaction, and abrasive wear is still the most important wear mode.

The rubber compound with 6 phr of TESPT has the least wear on the metal. When more TESPT is added, the wear of the metal remains unchanged. As the TESPT dosage increases, abrasive and corrosive wear proportion gradually changes. It is observed that the balance of the abrasive wear decreases, and the proportion of the caustic wear increases, reaching the maximum value of 20.7%. In the mixing process, abrasive wear is the main wear of the rubber compound to the metal. However, the corrosive wear caused by high-temperature steam still occupies a large proportion of the total wear. Therefore, it is very important to improve silica dispersion and reduce abrasive wear to protect the mixing chamber. Meanwhile, attention should be paid to the corrosion of the high-temperature water vapor on the metal.

## Figures and Tables

**Table 1:** Formulation.

Component (phr)	C1	C2	C3	C4	C5	C6	C7
NR	100	100	100	100	100	100	100
Silica115MP	60	60	60	60	60	60	60
ZnO	2	2	2	2	2	2	2
4020	2	2	2	2	2	2	2
SAD	2	2	2	2	2	2	2
TESPT	0	2	4	6	8	10	12
DPG	1.3	1.3	1.3	1.3	1.3	1.3	1.3
S	1.3	1.3	1.3	1.3	1.3	1.3	1.3
CZ	1.8	1.8	1.8	1.8	1.8	1.8	1.8

Natural Rubber (NR); Silica (Silica115MP); Silane Coupling Agent (TESPT); Zinc Oxide (ZnO), rubber Additives; Stearic acid (SAD); Rubber antioxidant (4020), N-1,3-dimethylbutyl-N’-phenyl-p-phenylenediamine; Accelerator diphenyl guanidine (DPG); Rubber vulcanization accelerator (CZ), N -Cyclohexyl-2-benzothiazole sulfenamide; sulfur (S).

**Table 2:** Traditional mixing process.

1.6L Hake Mixer, 80rpm, 75% FF		
Time	T (°C)	Ingredients
Masterbatch		
0:00	70	Polymers
0:40		Chemical, Silica
1:10		Silica
2:30	120	Sweep
4:00	135	Sweep, Sampleing
5:00	145	Discharge

The preparation process for the mixed rubber is shown in Table 2. Put in the cut NR first, and add the ingredients (except sulfur S and accelerator CZ) and half of the Silica115MP at 40 s. Add the other half of the Silica115MP at 1 min and 10 s. Sweep at 2 min 30 s and 4 min, and add the spilled compound to the mixer. Remove the rubber at 5 min. Then the rubber compound is pressed by a double-roll mill to obtain a rubber sample with a smooth and flat surface. Finally, it is cut with abrasive tools to provide the required pieces for the experiment [8,9,10].

**Table 3:** Settings of the RPA.

Stage	Frequency/hz	Temperature/°C	Time/Min	Strain	Test Items
1	0.1	60	5	0.28%	-
2	1	60	-	0.28–40%	$G^{\prime }(02)$
3	1	60	-	0.28–40%	$G^{\prime }(03)$
4	0.1	60/160/160	0/2.5/5	0.28%	-
5	1	60	-	0.28–40%	$G^{\prime }(05)$
6	1	60	-	0.28–40%	$G^{\prime }(06)$

**Table 4:** Dispersion images and value of rubber compounds with different TESPT phr.

Rubber Compounds	C1	C2	C3	C4	C5	C6	C7
Dispersion	5.42	5.98	6.51	7.47	7.56	7.49	7.53

**Table 5:** Silanization reaction indices of different compounds.

Rubber Compound	C1	C2	C3	C4	C5	C6	C7
Silanization reaction index	0	0.16249	0.30586	0.55879	0.54589	0.55969	0.56328
